# When less is more: the role of non-vitrectomized vitreous surgery in retinal diseases

**DOI:** 10.1186/s40942-025-00686-1

**Published:** 2025-08-01

**Authors:** Andrew D. Brown, Ahmed F. Shakarchi, Muhammad Z. Chauhan, Lindsay Chai-Chang, Daisy Alapat, Abdulrahman H. Badawi, Ahmed B. Sallam

**Affiliations:** 1 Department of Ophthalmology, Bernice and Harvey Jones Eye Institute, University of Arkansas for Medical Sciences, Little Rock, 72223 Arkansas USA; 2 Department of Pathology, University of Arkansas for Medical Sciences, Little Rock, Arkansas USA; 3 King Khaled Eye Specialist Hospital, Riyadh, Saudi Arabia; 4 Department of Ophthalmology, Ain Shams University, Cairo, Egypt

**Keywords:** Non-vitrectomized vitreous surgery, Pars plana vitrectomy, Limited vitrectomy, Epiretinal membrane, Vitreous preservation, Retinal detachment

## Abstract

Anatomically, the vitreous is not merely a transparent medium; it is a complex gel structure crucial for maintaining intraocular architecture and biochemical homeostasis. By minimizing disturbance to the vitreous, non-vitrectomized vitreous Surgery (NVS) which involves surgery with no or minimal removal of the vitreous gel, attempts to achieve the indication of the surgery while preserving the vitreous’ physiological functions, such as buffering intraocular oxygen tension and limiting the influx of inflammatory cells. This selective approach is especially relevant in younger patients with clear crystalline lenses, and in those with healthy peripheral retinas. In such cases, removing only the targeted portion of the vitreous gel or vitreoretinal interface, rather than performing a complete PPV, may reduce oxidative stress and inflammation, thereby translating to lower long-term complications such as cataract progression or peripheral retinal tears. NVS may lend itself to simple ERMs, vitreous opacities, diagnostic biopsies and localized superior rhegmatogenous retinal detachments but is not suitable for extensive vitreous or retinal pathology or advanced retinal detachment.

**Supplementary Information:**

The online version contains supplementary material available at 10.1186/s40942-025-00686-1.

## Philosophy

Non-vitrectomized vitreous Surgery (NVS) is founded on the principle of preserving the integrity of the vitreous body as much as possible. Unlike traditional pars plana vitrectomy (PPV), which often involves extensive vitreous removal, NVS aims to minimize vitreous manipulation to reduce associated complications and enhance recovery. By targeting specific problem areas while leaving the surrounding structure intact, NVS seeks to mitigate surgery related complications such as cataract progression and retinal breaks as well as reduce surgery time and cost.

Early interest in NVS was influenced by Margherio et al. in 1985, who observed a low rate of nuclear sclerosis (NS) in patients with short surgical times, increased infusion port distance from the lens, and techniques preserving the anterior vitreous behind the lens [1]. This premise continues to be strengthened by recent research demonstrating PPV increases intraocular oxygen tension, leading to lens fiber oxidation and subsequent cataract formation [2–5]. Additionally, induction of a complete posterior vitreous detachment (PVD), as is customary during PPV, has been shown to increase the likelihood of retinal breaks [6].

The underlying philosophy of NVS is to provide effective treatment for noncomplex vitreoretinal conditions while maintaining a ‘gentler’ impact on the eye’s natural anatomy, ultimately resulting in better patient outcomes and reduced postoperative complications [7].

## History and technique progression

### Initial developments

One of the first comprehensive studies of NVS was led by Saito et al. in 1999, in its use in treating ERMs [7]. Their surgical methodology involved creating only 2 sclerotomy sites, either 20- or 23-gauge, in the superotemporal and superonasal quadrants, without the use of an infusion port. A fiberoptic halogen light source and a microhooked needle were then introduced through the sclerotomies. The ERM was removed immediately with no prior vitreous cutting or other intraocular maneuvers.

Vitreous incarcerated at the sclerotomy sites was trimmed using microscissors before sclerotomy closure. No intraocular solutions were injected into any of the eyes, even in cases of mild hypotony, which was present in nearly all cases. In response to the hypotony highlighted in this previous study, Alvin Kwok and Dennis Lam proposed their “self-healing” sclerotomy technique in a letter to the editor [8]. Their method involved initiating the scleral tunnel with a crescent knife, followed by perforation of the inner sclera at the tunnel’s endpoint using a 20-gauge round-body hypodermic needle, replacing the conventional myringo-vitreal-retinal (MVR) blade. This needle was subsequently utilized for membrane peeling, eliminating the need for repeated entry through the sclerotomy.

### Refinements of technique

Building on the foundational work of Saito et al., subsequent studies by Sawa et al. introduced significant refinements to the NVS technique in 2006 [9]. Their approach incorporated the use of a 25-gauge vitrectomy system. This method offered superior control of postoperative hypotony due to the smaller sclerotomy size of the 25-gauge system, which is approximately one-fourth to one-third the size of the previously utilized 20-gauge sclerotomies. The transition from conventional sclerotomies to the trocar system also reduced the pressure exerted on the eye during instrument insertion, enhancing safety and precision. To further mitigate hypotony, some authors have advocated refilling the vitreous chamber with a mixture of 20% sulfur hexafluoride (SF6) and air when signs of hypotony are observed [10, 11].

Around the same time, endoillumination and microforceps technology advancements facilitated significant improvements in NVS instrumentation. Traditional halogen and metal halide light sources were replaced by stand-alone xenon systems, which provided superior illumination and enabled smaller port sizes in retinal surgeries [12]. Sakaguchi et al. capitalized on these developments by introducing a refined technique that employed newly designed microforceps and a 27-gauge chandelier probe [13]. The chandelier probe was anchored transconjunctivally at the superior pars plana, replacing the fiber-optic light probes used in earlier iterations of the procedure. This innovation further downsized the NVS approach, enhancing precision and minimizing invasiveness.

### Adapted techniques, limited vitrectomy

Adapted techniques, such as “limited vitrectomy,” gained popularity by incorporating principles of NVS with a standard 23- to 25-gauge, three-port PPV system [14–21]. These approaches involve removing a larger portion of the vitreous compared to NVS while deliberately preserving 3–4 mm of vitreous behind the lens or trimming the vitreous anterior to the equator without complete removal [21]. Efforts are made, when possible, to avoid disturbing the peripheral cortical vitreous and vitreous base [14]. Preserving the anterior vitreous provides a protective barrier against oxidative stress caused by oxygen free radicals. Additionally, some practitioners opt to avoid inducing PVD during limited vitrectomy, given association with retinal breaks and cataracts in conventional vitrectomy [6, 22]. though this risk has not been specifically reported in the context of NVS.

Limited vitrectomy has demonstrated success in removing vitreous opacities and vision-degrading floaters while minimizing the risk of post-operative cataract formation [19, 23]. Additionally, it has been used for ERM removal and macular hole surgery [14, 20, 24]. Limited vitrectomy has also been proposed for treatment of pseudophakic rhegmatogenous retinal detachment (RRD) in cases where a small gas fill would suffice as in detachments due superior retinal breaks. The technique involves posterior hyaloid induction and removal of the vitreous until the level of the vortex veins without vitreous base shaving followed by laser retinopexy and gas injection. In this context, it was hypothesized that non-extensive vitrectomy technique without vitreous base shaving sufficiently relieves traction to achieve reattachment while minimizing retinal trauma and decreasing the development of proliferative vitreoretinopathy (PVR) [18]. The authors postulated that the preserved hyalocytes secrete TGF-β and are believed to suppress retinal pigment epithelial proliferation, key events in PVR pathogenesis.

A more limited, 2-port, dry posterior vitrectomy has also been adapted for some selected cases of superior RRD. In this technique, a localized vitrectomy close to the main retinal break was performed with the 25-gauge vitrectomy, under light pipe illumination, without any infusion system. The purpose was to release any tangential vitreoretinal traction and to drain subretinal fluid, followed by 20% SF6 gas and a cryopexy for retinal tears [10]. Limited anterior vitrectomy has also been used in some challenging anterior segment surgeries, such as in phacomorphic glaucoma with a very shallow anterior chamber, where it decompresses the vitreous and deepens the anterior chamber, facilitating safer phacoemulsification​ [15, 16]. Moreover, limited vitrectomy has found application in delivery of subretinal gene therapies for inherited retinal dystrophies, such as retinitis pigmentosa and Leber congenital amaurosis, where its precision ensures atraumatic administration while still being able to maximize coverage area [25–27].

In macular hole repair, limited vitrectomy involves induction of a posterior hyaloid detachment centrally, typically posterior to the equator, followed by internal limiting membrane peeling and gas tamponade. Unlike conventional surgery, this approach avoids peripheral hyaloid separation and extensive vitreous removal. Authors have proposed that limited surgery is sufficient to address vitreomacular traction and achieve macular hole closure [20].

## Current indications for NVS

NVS has been employed across various clinical scenarios. Initially developed for treating ERMs [7, 9, 13, 28, 29], NVS has since been adapted for the application of tissue plasminogen activator for premacular hemorrhages [30, 31]. Transvitreal biopsy of choroidal lesions is also another application of NVS using 25- or 27-guage needle or vitrectors [32, 33]. Additionally, NVS has been utilized in the treatment of some selected cases of noncomplex macular hole [20] and some types of RRDs, particularly those with retinal breaks confined to superior retinal quadrants [10, 11].

## Clinical outcomes

Saito et al., reported successful resolution of ERMs in 21 eyes, with no statistically significant increase in NS compared to non-operated fellow eyes (*p* = 0.631) over an average follow-up period of 9.7 months [7]. Long-term outcomes published by Sawa et al. extended this data with 30 successful NVS ERM procedures (mean follow-up of 72.2 months) [34]. Visual acuity improved or stabilized in 96.7% of eyes, with no progression of NS (*p* = 0.836).

In a case series of 3 eyes, Wu et al. demonstrated resolution of the premacula hemorrhages in all cases while using their NVS membranotomy to allow the hemorrhage to drain into the posterior chamber [30]. Preoperative visual acuities were recorded as counting fingers in all cases. Postoperatively, in case 1, visual acuity improved to 20/40 at one day and 20/20 by three weeks. In case 2, visual acuity improved to 20/200 at one day, 20/25 at one month, and remained stable at 20/25 at 30 months. In case 3, visual acuity improved to 20/400 at one day, 20/120 at two months, and 20/50 at six months. The study also noted no complications or cataract progression during the follow-up period.

In their study using NVS for the application of tissue plasminogen activator for 3 eyes with premacular hemorrhage, Preoperative visual acuities ranged from 20/600 to 20/120, with final vision improving to 20/33, 20/13, and 20/10 [31].

When used for transvitreal biopsy of choroidal melanoma, NVS performed using a 25-gauge needle had better overall cellularity and diagnostic yield (100%) when compared to a smaller 27-gauge needle (60%) [32]. However, there was no difference in the yield by instruments gauge when biopsy was mainly for performed for molecular prognostic testing and only a small sample tissue biopsy was required for genetic studies. Regarding safety, studies on transvitreal biopsy of choroidal tumors reported no significant complications associated with the technique such as RRD or epibulbar tumor seeding [33].

Regarding the use of limited vitrectomy for RRD, Wibbelsman et al. achieved a primary anatomical success of 88.9% in their series of 27 eyes [18]. Caporossi et al. reported an 86% primary anatomical success rate at six months using their NVS “dry vitrectomy” technique across 12 eyes for macula-on RRD [10]. No intraoperative complications were observed, with the exception of two cases of re-detachment, which were attributed to inadequate postoperative positioning. In their larger follow-up study involving 20 eyes, an 85% anatomical success rate was achieved at six months [11]. These outcomes are comparable to previous studies, which have reported success rates of 75.5 to 80.8% for pneumatic retinopexy (PnR) [35–38] and to some extent the outcomes for conventional PPV of 86.1–98.7% [35, 39, 40].

### Efficiency and time savings

Studies have highlighted reduced operative times with NVS, primarily due to the minimal vitreous removal required, which naturally shortens the procedure [1, 28, 41]. Ferrari et al. documented a mean surgical time of 7.5 min for NVS compared to 16.5 min for PPV in the treatment of ERMs [28]. Similarly, in cases for RRD, NVS demonstrated an average duration of 8.6 ± 2.16 min [10]. This time efficiency minimizes patient exposure to intraocular interventions but also offers potential benefits such as cost savings and increased procedural capacity in clinical settings.

## Challenges

The practical limitations of NVS stem mainly from its narrow set of indications, as outlined earlier. Furthermore, one of the early concerns with NVS during the large 20-guage era was incomplete ERM removal. Saito et al. observed retinal bleeding in 12 out of 21 eyes, with visibility issues contributing to incomplete removal of membranes in 4 cases [7]. Sawa et al. reported a residual or recurrence rate of 33% over a mean follow-up period of 72.2 months [34]. This recurrence rate was notably higher than the 4–12% associated with traditional PPV. The residual or recurrent ERMs were postulated to result from the incomplete removal of ERM-causing cells and residual membrane fragments secondary to decreased intraoperative visibility from retinal surface bleeding during the membrane peel. Retinal surface bleeding, though not uncommon during PPV is another concern. This may have been exacerbated in earlier large-bore NVS procedures due to the associated hypotony caused by prolapse from the sclerotomy sites and the absence of infusion fluid [8, 42, 43]. Notably, surface bleeding, residual ERM, and hypotony have not been discussed as complications since 2005. Later, iterations of NVS by Caporossi and Peiretti addressed hypotony by refilling the vitreous chamber with a mixture of 20% SF6 and air [10, 11].

Questions remain about the risk of iatrogenic retinal breaks with limited to no vitreous removal in NVS. While vitreous incarceration within surgical instruments or in sclerotomy sites and postoperative development of PVD are recognized concerns with NVS, so far published clinical outcomes have demonstrated a low incidence of related retinal tear or RRD. Notably, even in conventional PPV, the value of extensive vitrectomy with vitreous-base shaving is debated: several ERM and macular-hole studies reported fewer postoperative tears when vitreous dissection was limited to the posterior pole and vitreous-base shaving was omitted [24]. Across published cohorts in which NVS was performed for vitreous opacities, ERM or macular-hole repair, RRD occurred in less than 2% [14, 20, 41] When NVS was used for primary RRD repair, redetachment rates were similarly low and congruent with conventional PPV repair rates [10, 18]. Wibbelsman et al. observed a single recurrent detachment due to a new retinal tear in 1 of 27 pseudophakic eyes.¹⁸ In another series, a redetachment case was attributed to suboptimal postoperative head positioning rather than to intraoperative factors.¹⁰.

## Discussion: NVS versus PPV

### NVS: a feasible alternative for selected cases

Preserving the vitreous, when possible, may offer significant clinical benefits. NVS’s minimally invasive approach may lend itself for simple ERMs and localized macula-on RRDs. Studies consistently demonstrate shorter surgical times with NVS, translating into reduced patient recovery periods and potential cost savings [10, 28, 41]. However, these potential advantages must be weighed against the limitations of the current evidence base, which is largely composed of small, retrospective studies.

Moreover, NVS’s ability to reduce complications such as cataract progression highlights its suitability for younger, phakic patients or those seeking a ‘gentler’ intervention. However, these benefits are possibly tempered by limitations, such as an increased recurrence rate of ERMs (33% in NVS vs. 4–12% in PPV), likely due to incomplete membrane removal or recurrence.

The success rates of NVS for macula-on RRDs have been shown to be not significantly inferior to established methods. Caporossi et al. reported an 85–86% primary anatomical success rate for NVS, aligning closely with success rates of 75.5–80.8% for PnR and 86.1–98.7% for PPV reported in previous studies. However, it is of note that this technique of limited vitrectomy in RRD would not be optimum in cases with inferior retinal breaks and retinal detachments. In these cases, more extensive vitrectomy is warranted for creating a bigger gas fill.

The presence or absence of a PVD may influence surgical planning in NVS and limited vitrectomy but does not contraindicate either approach. When a spontaneous PVD is present, traction is reduced, and membrane removal may be more straightforward. However, in cases without a PVD, surgeons may choose to safely induce one or proceed without induction. Both options have demonstrated acceptable safety profiles in published studies [6, 22, 44–46].

When considering NVS for RRD, case selection must include careful evaluation of break number, size, and location. NVS is most appropriate for single or few breaks confined to the superior quadrants, without PVR. Extensive inferior breaks, multiple breaks in multiple quadrants, large horseshoe tears, or any PVR grade C or worse are contraindications to NVS and should prompt standard PPV.

### PPV: the gold standard for complex pathologies

Conventional PPV continues to be indispensable for advanced retinal conditions. In cases of PVR, giant retinal tears, or severe RRDs, PPV’s comprehensive approach ensures superior anatomical and functional outcomes. It also allows for the simultaneous management of multiple pathologies, such as combined tractional and rhegmatogenous detachments, making it the cornerstone of vitreoretinal surgery.

However, PPV’s more invasive nature comes with a higher risk of complications, including increased oxidative stress leading to cataracts and a greater likelihood of retinal breaks and detachments.

### When to consider NVS

**Fig. 1:**
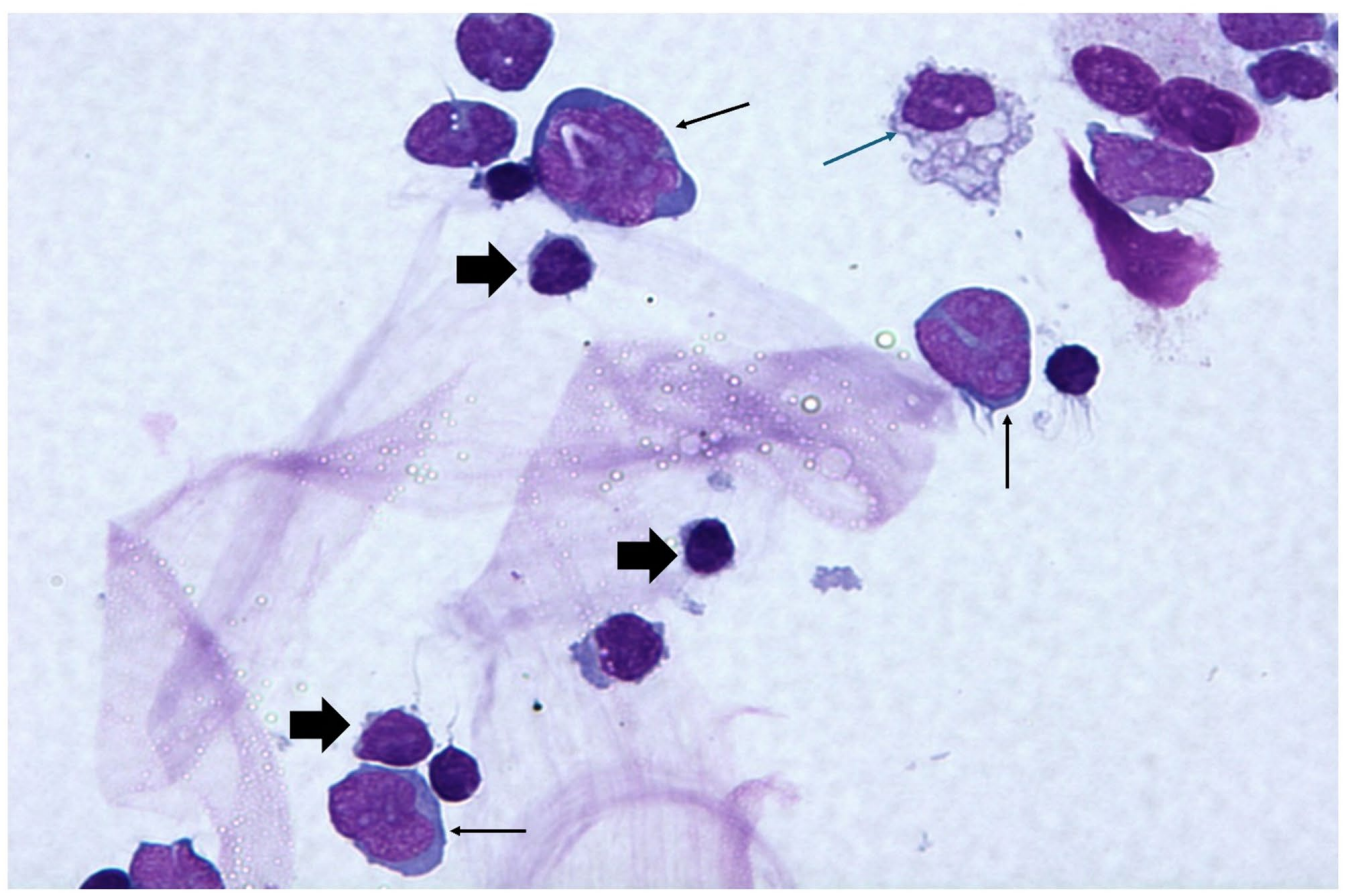
Diff-Quick stain, 50x magnification, showing large, atypical lymphoma cells with irregular nuclear contours with prominent nucleoli and moderate amount of cytoplasm (thin black arrow). Note the presence of small mature lymphocytes (thick black arrow) and a monocyte (thin black arrow) in the field. Further testing characterized B cell lymphoma with cells being kappa light chain restricted, positive for CD19/ CD20 and negative for CD5/CD10

In summary, the choice between NVS and conventional PPV should be guided by the complexity of the pathology and patient-specific factors:

- Consider NVS or its adapted limited vitrectomy technique for:
  - Localized ERMs.
  - Some cases of non-large primary macular holes.
  - Macula-on RRDs with superior retinal breaks.
  - Diagnostic vitreous biopsy (Supplementary Material S1, Fig. 1) or chorioretinal biopsy.
- NVS is not suitable for:
  - Advanced RRDs, especially with inferior retinal breaks, PVR or giant retinal tears.
  - Combined tractional and rhegmatogenous detachments.
  - Pathologies requiring extensive vitreous removal for optimal outcomes.

## Study limitations

We present a narrative review of the available studies, which are heterogeneous, with most being small, retrospective, and uncontrolled. These limitations introduce risks of biases including selection and recall biases. Due to the small number and variability of studies, meta-analysis was not possible.

## Conclusion: is NVS a feasible alternative?

NVS is not a direct replacement for PPV but it may be a useful addition to the vitreoretinal surgeon’s repertoire in some selected noncomplex vitreoretinal procedures. Future advancements in instrumentation and procedural adaptations may further expand its applicability. Although the limited vitreous excision inherent to NVS has raised concerns about iatrogenic retinal breaks, published series to date report a very low incidence of postoperative complications; nonetheless, long-term follow-up studies are still needed.

## Supplementary Information

Below is the link to the electronic supplementary material.

**Supplementary Material 1:** Limited vitrectomy, an adapted technique of non-vitrectomized vitreous surgery, used for obtaining undiluted vitreous biopsy under air in a patient with suspected intraocular lymphoma. Pathology results confirmed the diagnosis of lymphoma.

## Data Availability

No datasets were generated or analysed during the current study.
